# The Pharmacological Effects of Phenylephrine are Indirect, Mediated by Noradrenaline Release from the Cytoplasm

**DOI:** 10.1007/s11064-022-03681-2

**Published:** 2022-08-09

**Authors:** Mahmoud Al-Khrasani, David A. Karadi, Anna R. Galambos, Beata Sperlagh, E. Sylvester Vizi

**Affiliations:** 1 Department of Pharmacology and Pharmacotherapy, Semmelweis University, Nagyvárad tér 4., Budapest, 1089 Hungary; 2 Institute of Experimental Medicine, Eötvös Lóránd Research Network, Szigony utca 43., Budapest, 1083 Hungary

**Keywords:** Phenylephrine, Indirect action, Noradrenaline transporter, Noradrenaline release, α_1_-Adrenoceptor, Cytoplasmic origin

## Abstract

Phenylephrine (PE) is a canonical α_1_-adrenoceptor-selective agonist. However, unexpected effects of PE have been observed in preclinical and clinical studies, that cannot be easily explained by its actions on α_1_-adrenoceptors. The probability of the involvement of α_2_- and β-adrenoceptors in the effect of PE has been raised. In addition, our earlier study observed that PE released noradrenaline (NA) in a [Ca^2+^]_o_-independent manner. To elucidate this issue, we have investigated the effects of PE on [^3^H]NA release and α_1_-mediated smooth muscle contractions in the mouse vas deferens (MVD) as ex vivo preparation. The release experiments were designed to assess the effects of PE at the presynaptic terminal, whereas smooth muscle isometric contractions in response to electrical field stimulation were used to measure PE effect postsynaptically. Our results show that PE at concentrations between 0.3 and 30 µM significantly enhanced the resting release of [^3^H]NA in a [Ca^2+^]_o_-independent manner. In addition, prazosin did not affect the release of NA evoked by PE. On the contrary, PE-evoked smooth muscle contractions were inhibited by prazosin administration indicating the α_1_-adrenoceptor-mediated effect. When the function of the NA transporter (NAT) was attenuated with nisoxetine, PE failed to release NA and the contractions were reduced by approximately 88%. The remaining part proved to be prazosin-sensitive. The present work supports the substantial indirect effect of PE which relays on the cytoplasmic release of NA, which might explain the reported side effects for PE.

## Introduction

The binding of drugs to receptors is a necessary step in the production of molecular and cellular responses. Since Ahlquist’s discovery [1] that receptors that are sensitive to noradrenaline (NA) may be classified as α- or β-receptors, several studies have provided convincing evidence that α-adrenoceptors may be further divided into subtypes of α_1_ and α_2_. Further developments in pharmacology and molecular biology revealed additional heterogeneity and the existence of six α- and three β-adrenoreceptors. Pharmacological and radioligand binding assays of α_1_ receptors expressed in native cells identified three subtypes [2]. Molecular biological studies have provided convincing evidence that there are three genes encoding α_1a_-, α_1b_- and α_1d_-adrenoceptors that correspond to the α_1A_-, α_1B_- and α_1D_- receptors [3]. Several reviews have discussed α_1_-adrenoceptor agonists [3, 4], and textbook chapters focused on these agonists, such as Goodman and Gilman and Rang and Dale’s Pharmacology [5, 6], in which phenylephrine (PE) was canonised as a primary and directly-acting drug on α_1_-adrenoceptors. This classification was supported by the potency and intrinsic activity of PE on recombinant α_1A_ receptors [7, 8]. This subtype is involved in NA-induced vasoconstriction which leads to increased blood pressure and contraction of the longitudinal muscle in the vas deferens and lower urinary tract via stimulation [9].

Phenylephrine (PE), a direct-acting α_1_-adrenoceptor agonist, is primarily used clinically as a vasopressor of choice to prevent spinal anaesthesia-induced hypotension in healthy parturients undergoing caesarean delivery [10] and in the treatment of hypotension in surgical patients and patients with septic shock. PE is also widely used as a nasal decongestant and mydriatic agent.

However, certain unexpected observations have been reported in preclinical studies of PE, including α_2_-adrenoceptor-mediated presynaptic inhibitory actions on the release of NA [11–13] and acetylcholine [14]. Flavahan and McGrath [15] demonstrated simultaneous α_1_, α_2_, β_1_ and β_2_ adrenoceptor-mediated effects of PE in the cardiovascular system of pithed rats. β-receptor-mediated positive inotropic and chronotropic effects of PE were observed in atria isolated from guinea pigs and rabbits, while methoxamine did not exhibit these effects [16]. Similarly to guinea pig, in rat papillary muscles, the positive inotropic responses to PE were unaffected by the α_1_ antagonist prazosin but were antagonised by propranolol, which indicated a β-adrenoceptor-mediated action, and again methoxamine had no effect [17]. PE inhibits stimulation-evoked serotonin release from raphe nuclei via α_2A_ receptor activation [13], which is a characteristic target of NA.

Similar unexpected observations were also found in human experiments and clinical practice, such as PE production of a β-adrenoceptor-mediated action in the human forearm [18]. β-receptor-mediated immunosuppression in response to PE infusion was recently shown in humans, specifically, enhanced IL-10 and reduced proinflammatory cytokine production [19], which compromised host defence and increased susceptibility to infection. Adverse effects, including an increased heart rate, a typical β-adrenoceptor action and decreased blood pressure, have been reported even after eye-drop applications in a meta-analysis [20].

The release of NA in response to PE has been consistently observed, but the contribution of the indirect component of PE to the pharmacological effect was deemed negligible [11, 21] or claimed as an effect of α_1_-adrenoceptor that was distinct from the effects in other organs [22]. Other studies provided convincing neurochemical evidence that PE [23], in contrast to methoxamine and (−)-amidephrine [24], released NA in a [Ca^2+^]_o_-independent manner in vas deferens isolated from mice. Due to the canonical interpretation in the literature and the consequence of the prominence of textbook data that PE is a direct-acting α_1_-agonist, the authors neglected to discuss or prove that the NA released by PE might also be involved in its effects on smooth muscle. MVD is well-known to host different types of α-adrenoceptors located both on presynaptic membrane and smooth muscle [25, 26].

The present study provides the first neurochemical and functional evidence that the release of a considerable amount of NA in response to various concentrations of PE is prevented, and the contractile effects (fast and slow) on smooth muscle cells are also substantially attenuated by the inhibition of NAT.

## Methods

### Animals

Male NMRI mice (35–45 g) were purchased from Toxicoop Zrt. (Budapest, Hungary) and their vas deferens was used for recording contractions. Male CD1 mice weighing 28–45 g were obtained from Charles River (Budapest, Hungary) and housed in a local animal facility and used for release experiments. Animals were kept in groups in a temperature- and humidity-controlled room under a 12-h light/dark cycle and under standard conditions of laboratory animal housing. The experiments followed the guidelines of the Ethical Board of Semmelweis University (EC Directive 2010/63/EU). The Semmelweis University Regional and the Institutional Committee of Science and Research Ethics committees approved the experimental protocol (PE/EA/285-5/2020). Food and water were available ad libitum. The animals were slightly anaesthetised before the tissue preparations.

### Isolated Mouse Vas Deferens

Experiments on smooth muscle contraction were performed as previously described with slight modifications [27]. Briefly, vasa deferentia were removed from the mice, desheathed and suspended between an upper (ring) and a lower (straight) electrode in 5-ml organ baths containing Krebs solution (concentrations in mM: NaCl, 118.0; NaHCO_3_, 25.0; KCl, 4.7; KH_2_PO_4_, 1.2; glucose, 11.0; CaCl_2_, 2.5; and MgSO_4_, 1.2) aerated with a gas mixture of 95% O_2_ and 5% CO_2_. The upper end of the isolated organ was attached to a transducer using a thread and connected to a computer via an amplifier. The resting tension was adjusted to 0.1 g. Electrical field stimulation was applied. The parameters of the stimulation included trains (10 Hz with 20 rectangular impulses at a 1-ms pulse width, 9 V/cm; i.e., supramaximal intensity) repeated with 0.1 Hz (Stimulator 88, Grass Medical Instruments, Quincy, MA, USA).

### Experimental Paradigms

In experiments designed to assess the effects of test compounds in MVD muscle contractions, vasa deferentia were equilibrated under electrical stimulation for 20–30 min before drug administration. Next, electrical stimulation was stopped, and PE or NA was administered at different concentrations and left for 2 min before washing. Electrical stimulation was initiated again, and nisoxetine or prazosin was added to the organ bath and left for 15–20 min to equilibrate before adding PE or NA again without electrical stimulation. The temperature of the Krebs solution was kept constant using a thermoelectric device (Frigomix 2000, B. Braun, Germany). Similar protocol was followed when the effect of PE was determined in MVD from mice treated with reserpine or vehicle (5 mg/kg, i.p., 18 h) prior to the experiments.

### Release of [^3^H]Noradrenaline

The release of [^3^H]NA was measured using ex vivo preparations from the MVD. The experiments were performed in strict accordance with the European Directive (2010/63/EU) and the institutional guidelines. Mice were anaesthetised and decapitated. The vasa deferentia were quickly removed and placed in ice-cold Krebs solution. Release experiments were performed as previously described [23]. Briefly, small pieces of the preparations were incubated in 1 ml of Krebs solution containing 5 μCi/ml [^3^H]NA for 45 min. After incubation, the preparations were transferred to micro tissue chambers and continuously perfused with Krebs solution at a rate of 0.5 ml/min. After 45 min of perfusion, samples were collected every 3 min and assayed for radioactivity. During the release experiments, Krebs solution contained 10 µM pargyline in order to inhibit the production of NA metabolites [28]. The total radioactivity released from the tissue and collected in the superfusate was accepted as the amount of [^3^H]NA released [29] [30]. In our earlier study the release of radioactivity as [^3^H]NA and its metabolites was confirmed by high pressure liquid chromatography combined with radiochemical detection [23].

The residual radioactivity in the preparations was extracted at the end of the experiments using 1 ml TCA (10%) for 120 min and measured in an aliquot (0.1 ml) of the supernatant. The radioactivity present in the tissues and samples was measured using a Packard-Canberra TR 1900 liquid scintillation counter, and the amount of [^3^H]NA content was normalised to the Bq/g of the tissue.

A computer program was used to calculate fractional release (FR). The measured radioactivity in the samples was calculated as the fractional release using a custom-made script

$$FR\% = \frac{released\, tritium\, in\, Bq/g \times 100}{{tritium\, in\, Bq/g\, in\, the\, tissue \,at\, the\, time\, of\, sample \,measurement}}$$

The effect of axonal (field) stimulation on release was evaluated as the total release of radioactivity over resting release (FRS_1_). The measured release during the first FRR_1_ (average resting release measured during the first and second collection periods) was considered as the internal standard. Unless otherwise stated, the drugs were administered as indicated in the experiments, and the ratio of FRR_1_ (average resting release fractions during the 1st and 2nd collections) and FRR_2_ (average resting release fractions during the 13th and 14th collections) (FRR_2_/FRR_1_) were calculated and used to demonstrate the effects on resting release. The effect of drugs on resting release was evaluated as the ratio of the area under the curve (AUC) of the total release of radioactivity during the first and second periods, as indicated (FRR_2_/FRR_1_).

Field stimulation-induced release of [^3^H]NA is tetrodotoxin (TTX)-sensitive and [Ca^*2*+^]_o_-dependent, which suggests a neuronal and vesicular origin of the release [31]. In contrast, the release was of cytoplasmic origin under resting conditions. In the vas deferens preparations the release of NA in response to PE was [Ca^2+^]_o_-independent in our experiments. For experiments in which calcium was removed, 1 mM EGTA was added to the Krebs solution, and the tissue was exposed to this modified Krebs solution throughout the experiments.

In a distinct set of experiments, mice were pre-treated with reserpine (5 mg/kg, i.p., 18 h) prior to the experiments.

Unless otherwise indicated, all experiments were performed at 37 °C in Krebs solution (pH 7.4). The same conditions were used during the recording of smooth muscle contractions, and the solution was continuously saturated with 95% O_2_ and 5% CO_2_.

### Reagents

Levo-[7-^3^H]-noradrenaline (specific activity = 20 Ci/mmol) was purchased from American Radio-labeled Chemicals (St. Louis, MO, USA). The following drugs were used: prazosin HCl (RBI), reserpine (Tocris Bioscience, Bristol, UK) nisoxetine HCl and cocaine HCl (Sigma-Aldrich, Budapest, Hungary). All other chemicals were obtained from Sigma-Aldrich (Budapest, Hungary).

### Data Analysis

In case of all the quantifications and data analysis, the analyst was blind to the origin of the data during statistical analysis. All the group data subjected to statistical analysis had a minimum of n = 5 of independent values per group. Group sizes are shown in each figure. Statistical analyses were performed using GraphPad Prism 8.01 software (San Diego, CA, USA). Normality was tested by Shapiro–Wilk test and based on the result, parametric or nonparametric tests were used. In contraction experiments with isolated mouse vas deferens, the area under the curve (AUC) values of the 2 min time period following PE or NA administration were calculated as the integral of the contraction curve relative to the baseline. When multiple comparisons were necessary, one-way ANOVA followed by Tukey’s post hoc test was used. Post hoc tests were run only if F achieved p < 0.05. Significant differences between two groups were analysed using two-tailed paired t-test. p < 0.05 was considered as statistically significant.

## Results

### Effects of Field Stimulation and Phenylephrine on Contractions and [^3^H]NA Release from the Vas Deferens

**Fig. 1:**
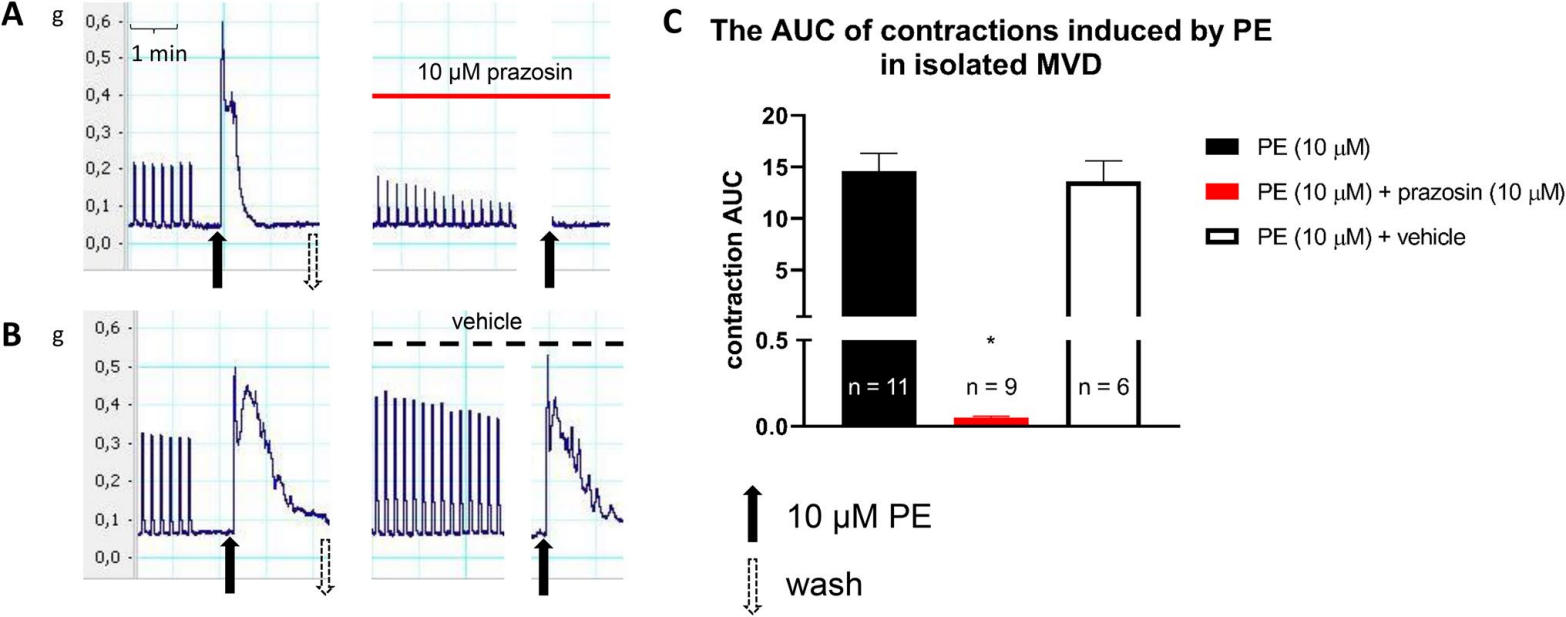
Effect of prazosin (10 µM) on electrical field stimulation or PE-induced contractions in isolated mouse vas deferens. Contractions induced by PE on mouse vas deferens in the presence of prazosin (**A**) versus vehicle (**B**). The organs were allowed to equilibrate under electrical stimulation (trains of 10 Hz with 20 shocks were delivered at 0.1 Hz) for 20–30 min before PE administration. Next, the organ bath was washed out, and the organs were equilibrated once more in the presence of prazosin for 15–20 min. The effect of PE is presented as AUC values (**C**), which were calculated as the integral of the contraction curve relative to the baseline of the 2 min period for each contraction. The AUC values are presented as the mean ± S.E.M. (**C**). *: significant difference versus control. Gaussian distribution was assumed following ns. Shapiro–Wilk test (alpha = 0.05). The significance levels were determined by one-way ANOVA followed by Tukey’s post hoc test

Consistent with previous studies [32, 33], a biphasic contractile response of the vas deferens to trains of electrical field stimulation was observed. PE produced a fast contraction, followed by the maintenance of smooth muscle contraction, in a concentration-dependent manner. Figure 1 shows that prazosin inhibited field stimulation and PE-induced contractions (Fig. 1A, C). The AUC values for the PE-induced contractions are shown in the presence of the postsynaptic α_1_-antagonist prazosin and vehicle (Fig. 1C).

In experiments designed to determine the effect of stimulation on the release of NA, after the preparations were loaded with [^3^H]NA, the average uptake of radioactivity was 1,001,280 ± 47,956 Bq/g (n = 24), and the average resting release during a 3-min collection period (FRR_1_) was 1.10 ± 0.11% of the total radioactivity (n = 8). Resting release was maintained: the FRR_2_/FRR_1_ was 0.94 ± 0.08 (n = 6) after 21 min elapsed between the two measurements. FRR_1_ was used as the internal standard. Electrical stimulation (10 Hz, 20 shocks) significantly enhanced the release of radioactivity at the top of the resting release (S_1_ = 61,553 ± 9724 Bq/g or 1.70 ± 0.25% of the total radioactivity) (n = 8, p < 0.05).

When the preparations were loaded with [^3^H]NA in parallel experiments the uptake of radioactivity was significantly lower in tissues in which 3 μM PE was added to the loading Krebs solution (755,000 ± 45,000 vs. 124,000 ± 43,000 Bq/g, p < 0.05, n = 6–6). This effect of PE is due to its substrate activity preventing [^3^H]NA for uptake by NAT.

**Fig. 2:**
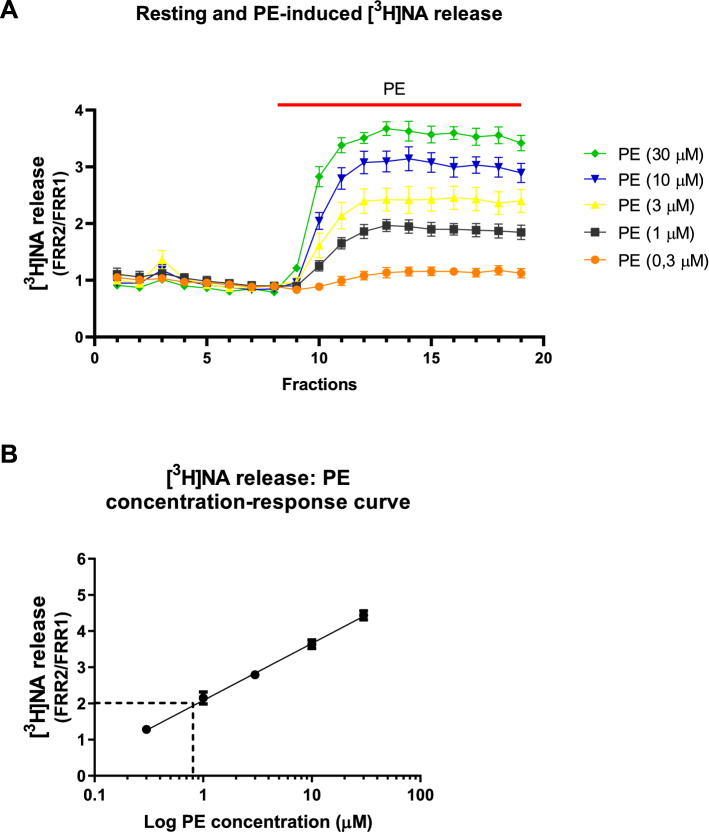
The resting release of [^3^H]NA induced by PE in a concentration between 0.3 and 30 µM in mouse vas deferens preparation. The release was measured as described in Methods. The preparation was stimulated with supramaximal voltage (10 Hz, 20 shocks) at third fraction. PE was added at different concentrations as indicated. The mean FRR values from fraction 1–8 in each group were compared with the FRR values from fraction 12–19 in that group using paired t-test. PE produced significant increase in NA release in concentrations 0.3, 1, 3, 10, 30 µM (p < 0.05) (**A**). Note the effect of PE on [^3^H]NA release is maintained. The concentration of PE is recorded against the effect on resting release (**B**). The dashed line indicates where the concentration of PE was able to double the extracellular concentrations of NA (approx. 0.8 µM). For calculations of FRR1 and FRR2 see Methods. n = 6 for each group

PE increased the resting release of [^3^H]NA in a concentration-dependent manner (Fig. 2A). The release was maintained as long as the tissue was exposed to PE. In fact, PE still significantly increased the release at concentrations as low as 0.3 μM (FRR_2_/FRR_1_ = 1.19 ± 0.07 (n = 6, p < 0.05) (Fig. 2A), and 0.8 µM PE was required to double the concentrations of NA (Fig. 2B) in the extracellular space.

**Fig. 3:**
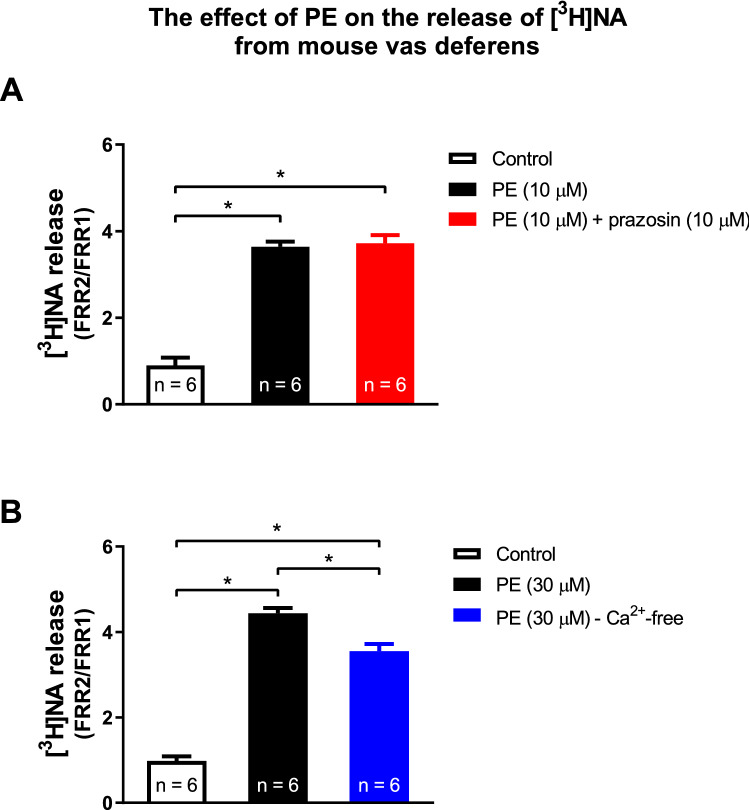
Prazosin failed to affect the PE-induced release of [^3^H]NA (**A**), the effect of PE is external calcium independent (**B**). The release was measured as described in Methods. Prazosin was added into the Krebs solution from the 6th fraction and kept in the solution throughout the experiment. PE was added from the 8th fraction. Values are presented as mean ± S.E.M. *: significant difference between groups. Gaussian distribution was assumed following ns. Shapiro–Wilk test (alpha = 0.05). The significance levels were determined by one-way ANOVA followed by Tukey’s post hoc test

Although prazosin failed to affect PE-induced NA release (Fig. 3A), it inhibited both fast and slow PE-evoked contractions (Fig. 1A, C) which indicated the role of α_1_-adrenoceptors in smooth muscle contractions. Similar to our earlier findings [4], the PE-evoked release was [Ca^2+^]_o_-independent (Fig. 3B), which indicated that the release was not vesicular in nature.

### Evidence of Cytoplasmic Release of NA in Response to Phenylephrine via the Transporter, Effects of Nisoxetine and Cocaine

**Fig. 4:**
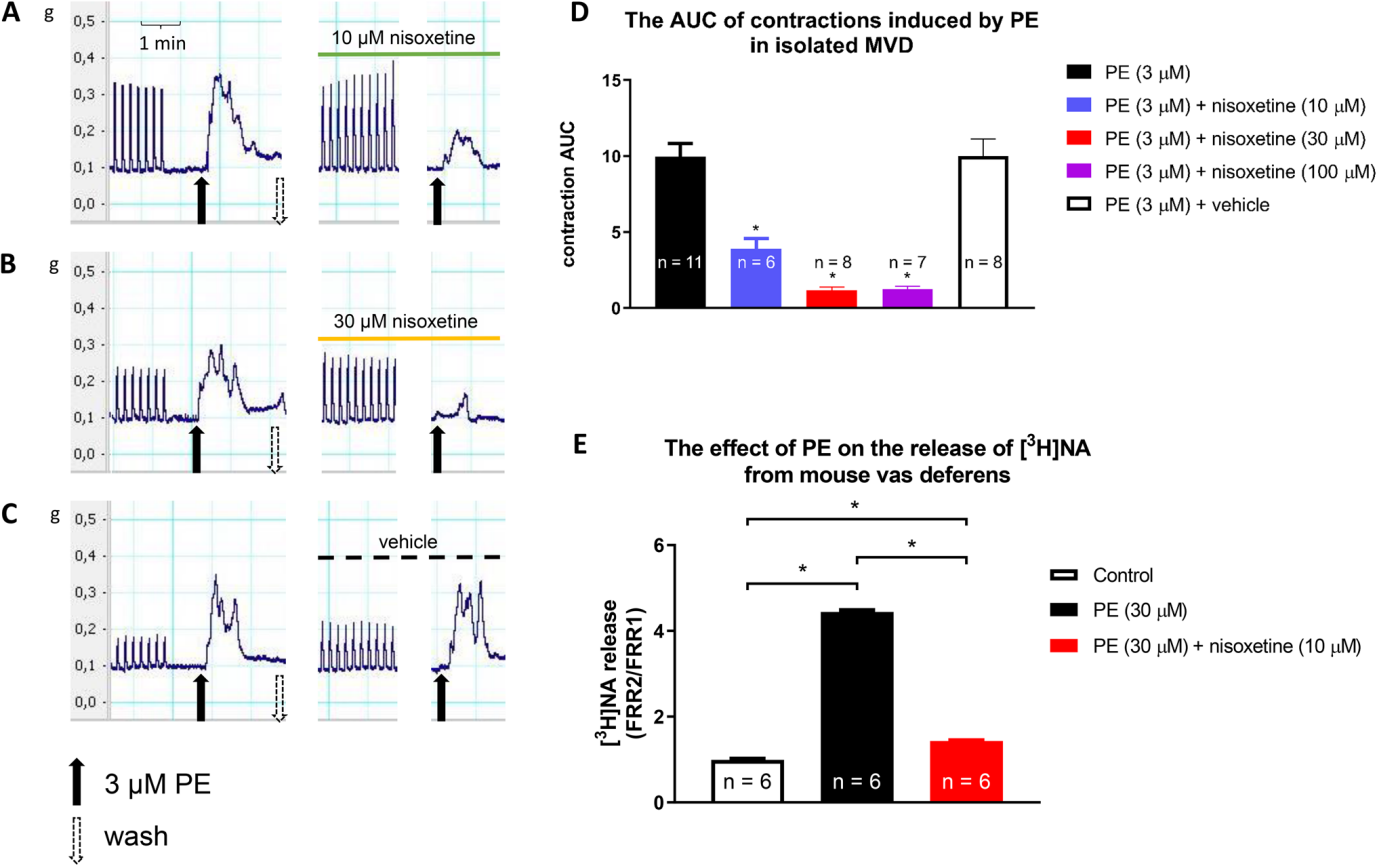
The effect of nisoxetine on electrical field stimulation or PE-induced contractions in isolated mouse vas deferens. Contractions induced by PE on mouse vas deferens in the presence of 10 μM nisoxetine (**A**), 30 µM nisoxetine (**B**) or vehicle (**C**). The organs were allowed to equilibrate under electrical stimulation (trains of 10 Hz with 20 shocks were delivered) for 20–30 min prior to PE administration. Next, the organ bath was washed out, and the organs were equilibrated once more in the presence of nisoxetine for 15–20 min. The effect of PE is presented as AUC values (**D**), which were calculated as the integral of the contraction curve relative to baseline of the 2 min period for each contraction. The AUC values are presented as the mean ± S.E.M., n = 6 for nisoxetine (10 or 30 µM) or vehicle; n = 14 for control (**D**). Nisoxetine was present in the Krebs solution from the 6th fraction throughout experiment and inhibited the release of [^3^H]NA induced by PE (**E**) measured at 13th and 14th collection periods. For method see legend of Fig. 2E. *: significant difference versus control or between groups as indicated. Gaussian distribution was assumed following ns. Shapiro–Wilk test (alpha = 0.05). The significance levels were determined by one-way ANOVA followed by Tukey’s post hoc test

**Fig. 5:**
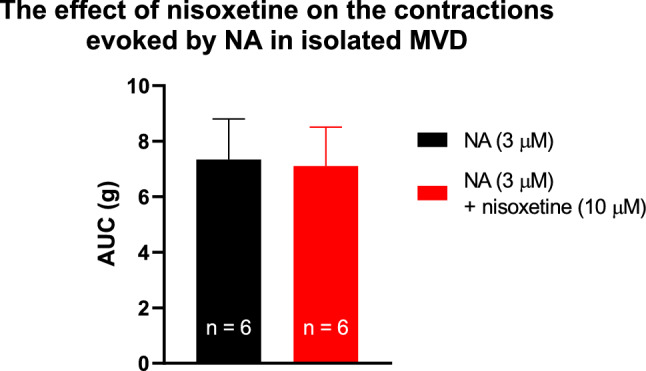
Effect of nisoxetine (10 μM) on NA-induced contractions in isolated mouse vas deferens. The effect of noradrenaline is presented as AUC values which were calculated as the integral of the contraction curve relative to the baseline of the 2 min period for each contraction. The AUC values are presented as the mean ± S.E.M., p = 0.7602 (ns). The significance level was determined by two-tailed paired t-test

**Fig. 6:**
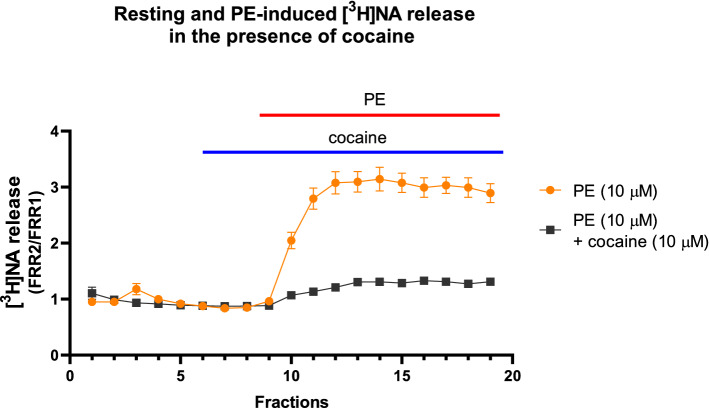
The resting release of [^3^H]NA induced by 10 μM PE in mouse vas deferens preparation in the presence or absence of 10 μM cocaine. The release was measured as described in Methods. The preparation was stimulated with supramaximal voltage (10 Hz, 20 shocks) at third fraction. Cocaine was added into the Krebs solution from the 6th fraction and kept in the solution throughout the experiment. PE was added from the 8th fraction. For calculations of FRR1 and FRR2 see Methods. Statistical analysis was made using two-way ANOVA followed by Tukey’s post-hoc test, the analysis was made between fractions 10–19, n = 6 for each group

Monoamine transporters play a pivotal role in altering the concentrations of monoamines in the extracellular space surrounding the axon terminals via an uptake mechanism. Nisoxetine is a selective inhibitor of NATs that prevents the reuptake of NA and increases the extraneuronal concentration of NA once released. Therefore, we studied the effect of PE when NAT was inhibited to identify the mode of action. Nisoxetine (10 µM) substantially inhibited PE from eliciting smooth muscle contraction (Fig. 4A, B, D) and completely prevented PE-induced NA release (Fig. 4E), supporting the critical role of NAT in this effect. PE (3 µM) produced contractions of AUC values of 10.17 ± 1.21 and 3.91 ± 0.65 or 0.86 ± 0.23 in the presence of 10 µM or 30 µM nisoxetine, respectively (Fig. 4D). Furthermore, nisoxetine failed to fully inhibit PE-evoked contractions, even at concentrations as high as 100 µM (Fig. 4D). This indicates that, at higher concentrations nisoxetine showed a ceiling effect in inhibiting the contractions. The finding that when NA release was inhibited by nisoxetine, the contractions that remained (~ 12% of the control) could be inhibited by prazosin, may represent the direct effect of PE on α_1_-adrenoceptors expressed on smooth muscle. The vehicle failed to affect PE-evoked contractions (Fig. 4C, D). Nisoxetine did not affect NA-induced smooth muscle contractions, which excluded its postsynaptic effects (Fig. 5). Cocaine is a nonselective monoamine transporter inhibitor that also significantly attenuated the release of NA in response to PE (Fig. 6).

### Effects of Phenylephrine on Contractions of the Vas Deferens When the Vesicular Storage of NA is Impaired

**Fig. 7:**
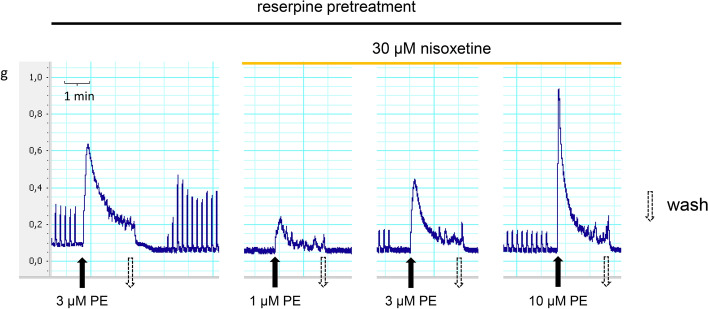
Representative figure of the concentration-dependent contractile effect of PE on smooth muscle under condition in which both vesicular and PE-induced transporter-mediated release of NA are impaired by reserpine pre-treatment and nisoxetine, respectively. The reuptake of NA is also inhibited by nisoxetine contributing to loss of releasable content of vesicles. Reserpinized mouse vas deferens (5 mg/kg i.p. 18 h). Note, the size of contractions induced by train (10 Hz, 20 shocks) at 0.1 Hz reduces by time and PE is still able to contract smooth muscle in a concentration-dependent manner. Take into account that smooth muscles to NA and PE (see Table 1) are supersensitive

Vesicular monoamine transport (VMAT2) regulates the storage and exocytotic release of monoamines [34]. Reserpine binds to vesicular transporters irreversibly [35] and prevents cytoplasmic NA from being stored in vesicles. Therefore, reserpine pre-treatment (5 mg/kg i.p. 18 h) was used to reduce or exclude the possible role of exocytotic NA in the effects of PE on contractions. Under the condition when the vesicular origin of NA was reduced or ruled out and reuptake of NA from the extracellular space was inhibited the responses to PE were potentiated in a prazosin-inhibition susceptible manner (Table 1). When vesicular storage of NA was impaired by reserpine-pre-treatment, and the NAT was inhibited by nisoxetine concomitantly, PE always retained some intrinsic activity in a concentration-dependent manner (Fig. 7.). Consistent with these findings, reserpine pre-treatment failed to prevent the positive inotropic effect of PE [22], and the responses of vas deferens to NA and methoxamine were potentiated 25-fold and fivefold, respectively, after 6-hydroxydopamine treatment [36] and surgical denervation [37].

**Table 1:** The effect of pre-treatment with or without reserpine on PE or NA-induced contractions in isolated mouse vas deferens

	AUC ± SEM (g)	
	PE (3 µM)	NA (3 µM)
No pre-treatment with reserpine		
Control^a^	11.35 ± 0.94 (n = 8)	9.46 ± 1.33 (n = 8)
Pre-treatment with reserpine		
Control^a^	20.22 ± 1.35^b^ (n = 9)	19.74 ± 1.79^b^ (n = 9)
Control^a^	21.78 ± 3.88 (n = 6)	N.D
Prazosin 10 µM	0.073 ± 0.018^c^ (n = 6)	N.D
Control^a^	21.27 ± 2.77 (n = 6)	N.D
Nisoxetine 10 µM	12.39 ± 2.74^c^ (n = 6)	N.D
Control^a^	18.04 ± 4.29 (n = 6)	N.D
Nisoxetine 30 µM	6.91 ± 1.69^c^ (n = 6)	N.D

The effect of 10 µM prazosin, 10 µM nisoxetine or 30 µM nisoxetine on PE-induced smooth muscle contractions. The organs were obtained from reserpine or no treatment (5 mg/kg ip.; 18 h prior to the experiment) animals. These preparations were allowed to equilibrate under electrical stimulation (trains of 10 Hz with 20 shocks were delivered) for 20–30 min prior to PE administration. Next, the organ bath was washed out, and the organs were equilibrated once more in the presence of prazosin or nisoxetine for 15–20 min. The effect of PE or NA is presented as AUC values which were calculated as the integral of the contraction curve relative to baseline of the 2 min period for each contraction. The AUC values are presented as the mean ± S.E.M

^a^Obtained from the first contraction induced by PE or NA

^b^Significant difference versus vehicle pre-treated

^c^Significant difference versus control. Gaussian distribution was assumed following ns. Shapiro–Wilk test (alpha = 0.05). The significance levels were determined by one-way ANOVA followed by Tukey’s post hoc test in the experiments assessing the effect of reserpine treatment on the contractions induced by PE or NA. Whereas, two-tailed, paired t-test was used in the experiments with prazosin or nisoxetine

### The Release of [^3^H]Noradrenaline from Preparations Following Reserpine Pretreatment

**Fig. 8:**
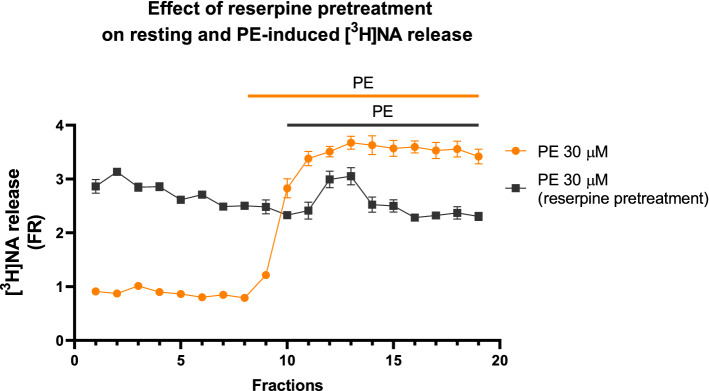
The resting release of [^3^H]NA induced by 30 µM PE in mouse vas deferens preparation of animals pre-treated with intraperitoneal reserpine (5 mg/kg i.p. 18 h). The preparation was not stimulated. For calculations of FRR1 and FRR2 see the Methods section. n = 5. The release of [.^3^H]NA in response to PE was taken from Fig. 2A. Statistical analysis was made using two-way ANOVA followed by Tukey’s post-hoc test, n = 5–5. In each fraction except for 10 and 1, NA release between the two depicted groups was significantly different (p < 0.05)

The release of [^3^H]NA was measured in vas deferens tissue prepared from animals untreated and pre-treated with reserpine in parallel experiments. While the uptake of [^3^H]NA was significantly lower in preparations dissected from reserpine-treated (5 mg/kg i.p. 18 h) than in controls (118,000 ± 8,620 Bq/g treated vs. 784,500 ± 30,608 Bq/g in the control, p < 0.05, n = 5–5) the resting release was significantly higher in these preparations (Fig. 8). PE produced a transient increase (Fig. 8). Although PE failed to exert its excessive effect on NA release to the same extent as in the control preparations, it still produced prazosin inhibitable contractions of a larger magnitude than the control experiments (Table 1). Spontaneously active contractions of the smooth muscle in reserpine treated preparations observed in few experiments are attributable to low NA content [37], the high spontaneous resting release of NA (Fig. 7) and the supersensitivity of the smooth muscle to NA (Table. 1).

## Discussion

PE has been reported to exhibit unexpected pharmacological effects in preclinical studies [12, 15–17] and clinical treatments [18–20], which are characteristic of α_2_- and β-adrenoceptor stimulation. These actions are not easily explained by a direct α_1_-adrenoceptor-mediated response to PE, a drug very frequently used in medical practice, which was shown and concluded in the studies published with several recombinant and native assays [2, 38–40] as typically representative of α_1_-agonists. Accordingly, PE is classified [3] as a purely directly-acting sympathomimetic in the overwhelming majority of publications [41, 42] and textbooks. The textbook classification of PE does not exclude the possibility that a directly-acting α_1_-agonist is able to release NA stored in axon terminals. This indicates that this release does not contribute to the response of effective cells. However, our data, suggest that this is not the case.

Our earlier study obtained neurochemical evidence, which indicated that the selective α_1_-adrenoceptor agonist PE [4, 23], but not methoxamine or (−)-amidephrine [4], both are accepted as α_1_-agonists, released NA from isolated vas deferens preparations in a [Ca^2+^]_o_-independent way.

The present study used one of the most useful ex vivo preparations for studying noradrenergic neurochemical transmission, isolated vas deferens, and showed that PE acts indirectly and its action is attributable to its NA-releasing capacity. Consistent with our earlier observations, PE released NA in a concentration-dependent manner (Fig. 2). The effect of PE on NA release is [Ca^2+^]_o_-independent [4] (Fig. 3B), which suggests that the release of NA is of non-vesicular origin. It is known that under physiological conditions, transmitters stored in vesicles are released in response to action potentials after extraneuronal Ca^2+^ enters the nerve terminals, but [Ca^2+^]_o_-independent release at rest is non-exocytotic, due to the reversal of the transporter [43]. Monoamines (NA, dopamine, and serotonin), phenylethylamine [44], and amphetamines [45] are translocated by transporters through the plasma membrane, which results in an efflux of transmitters residing in the cytoplasm [45–47]. PE is also known to be the substrate of the uptake mechanism [48] and the reverse mode of transporter function produced by extraneuronal substrates can be prevented by the inhibition of transporters [45]. Indeed, nisoxetine, a selective NAT inhibitor impeding translocation of the substrate prevented both PE-induced NA release (Fig. 4E) and substantially attenuated smooth muscle contractions (Fig. 4A, B, D), and did not influence the contractile effect of exogenous NA on smooth muscle (Fig. 5). Meanwhile cocaine, a nonselective transporter inhibitor, also reduced PE-induced NA release (Fig. 6), strongly indicating role of NAT in the α_1_-adrenoceptor activity of PE. These results provide convincing evidence that the contractile effect of PE on smooth muscle was due to NA released from noradrenergic axon terminals and that NAT played a critical role in the cytoplasmic release of NA, an effect mediated via transporter reversal independently on axonal activity and represents an analog type of signal transmission described in the central nervous system [44]. The distinct role of the substrate property of PE in its indirect effects is supported by the fact that methoxamine, an α_1_-adrenoceptor agonist but unlike PE, is not a substrate of NAT [48] and does not release NA [49].

The role of ATP released from the nerve terminals [29, 50, 51] in sympathetic transmission, specifically in contracting visceral and vascular smooth muscles, is widely accepted [33]. Furthermore, the observations of Westfall et al. [52] that ARL 67156, an ecto-ATPase inhibitor enhanced the responses of the vas deferens to various activations also indicate the possible involvement of ATP. The fact that ATP and NA are co-stored in synaptic vesicles [53] [54] and are co-released from sympathetic nerve terminals in response to axonal stimulation [55] supports the role of presynaptic ATP. Nevertheless, the contractile effect of PE is due to the non-vesicular and [Ca^2+^]_o_-independent release of NA (Fig. 3B) from the cytoplasm, which may indicate that the role of ATP of presynaptic origin in the effect of PE can be ruled out. Whereas, the role of postsynaptic ATP released in response to α_1_-adrenoceptor activation by NA [31] remains to be studied.

Given the fact that under conditions in which vesicular release, due to reserpine-pre-treatment, or cytoplasmic origin of NA by nisoxetine, were excluded, PE always retained some intrinsic activity in a concentration-dependent manner (Fig. 7) and contracted smooth muscle in a prazosin-inhibitable manner (Table 1) indicates that this effect of PE might have been mediated directly via α_1_-receptors and could have been estimated in binding studies with recombinant methods.

The interaction between PE and NAT described in the present paper, in which NAT in operation is eo ipso needed for PE to be able to release NA raises a clinically important question. As far as the future clinical practice is concerned, taking into account our observations that PE, the substarate of transporters inhibited NAT function, brings attention to the possibility of interactions. In patients who are undergoing surgery and have been chronically treated with tricyclic antidepressants, the application of PE to prevent hypotension during spinal anaesthesia may be less effective because the release of NA due to impairment of NAT function is prevented. This interaction seems to be similar to those observed in experiments shown in Fig. 4.

There has been a major change in the use of PE (instead of ephedrine) in spinal anaesthesia for the treatment of hypotension during surgery [56]. PE is sometimes preferred because of its selective α_1_ action. The NA-releasing property of PE described in the present study and its off-target effects described in several studies may be risk factors that compromise host defence and increase susceptibility to infection, which are strongly related to its effects on β-adrenoceptors expressed on immune cells [57], resulting in an immunosuppressive state [19, 58].

**Fig. 9:**
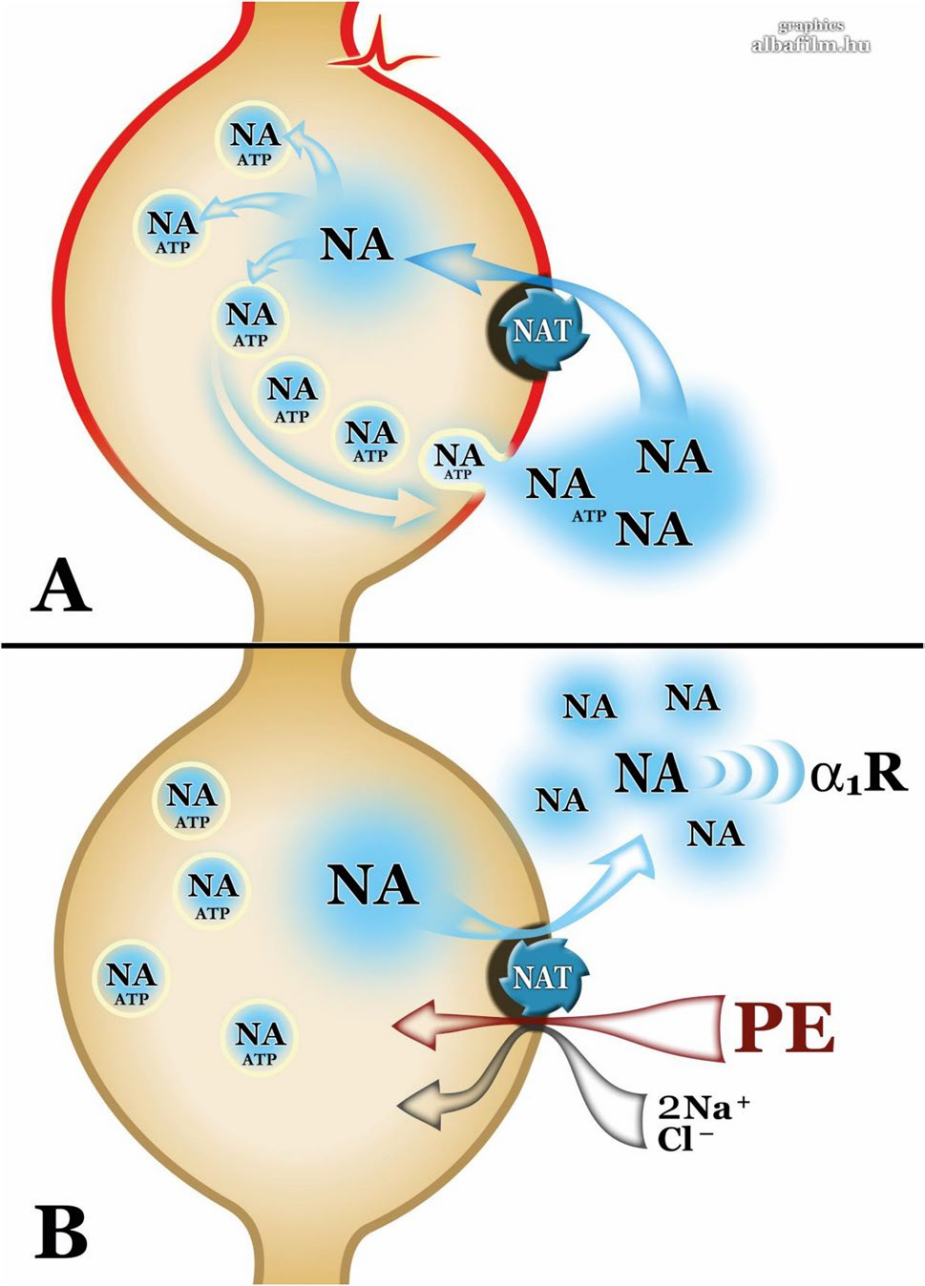
Mode of action of PE. Role of NAT in vesicular (**A**) and cytoplasmic (**B**) release of NA. **A** NAT controls the temporal and spatial action of released NA by taking back from the extracellular space and NA reused for refilling vesicles. **B** PE is the substrate of NAT and by means of NAT transported into the cytoplasm together with two Na^+^ and one Cl^−^ ions [45] followed by a counter movement of NA into the extracellular space where it acts on α_1_-adrenoreceptors. The effect is large in extracellular concentrations, it does not require axonal activity and Ca^2+^ influx, and hence it is termed non-exocytotic release from noradrenergic boutons without making synaptic contacts [62]. The smooth muscle cells are equipped with highly sensitive α_1_-adrenoceptors, this type of non-synaptic receptors are the target of drug treatment [63]. Nisoxetine, a selective NAT inhibitor, inhibits the uptake of NA released in response to action potential (see **A**) or prevents PE from entering the nerve terminal and the subsequent NA release (**B**)

In summary, although the various effects of PE have been narrowly constrained to α_1_–adrenoceptors in existing textbook data, we conclude that PE is not a selective, directly-acting α_1_-adrenoceptor agonist. Rather, its pharmacological effects on α_1_-adrenoceptors and several unexpected side effects that are characteristic of α_2_- and β-adrenoceptor activation observed in pharmacological experiments and clinical practice are due to the cytoplasmic release of NA mediated by the off-target effect of PE on NAT (Fig. 9). However, among the compounds studied with respect to α_1_-adrenoceptors, significant deviation between their functional and binding affinities was also shown [40, 59, 60] calling attention that homogenate radioligand binding studies in recombinant systems may present a bias and may not predict the real pharmacological profiles of the drug studied [40]. Therefore, these type of studies should be translated to therapeutically relevant native tissues equipped with receptors [40] including native-like molecular environment [61], and more importantly using intact animals. In addition, in studies in which PE used as standard reference of α_1_-agonists, and carried out in tissues containing some source of NA, that is the endogenous native agonist of PE, at potency and efficacy estimation its NA-releasing capacity should be taken into account.

## Data Availability

The datasets generated and analysed during the current study are available from the corresponding author on reasonable request.
